# The *Dental Digest*

**Published:** 1895-04

**Authors:** 


### THE "DENTAL DIGEST."

WE quote the following interesting piece of information from the February number of this new journal:

"The paper on the 'Relative Penetrating Power of Coagulants,' by Dr. James Truman, has the double distinction of being published also in the *Dental Cosmos.* The paper, with numerous experiments, was prepared to show the penetrating power of coagulants rather than diffusion. After taking finely attenuated tubes of glass and *partially filling them with albumen and glycerin*, various coagulating and non-coagulating agents were applied to the surface of the mixed liquid in the tubes. . . . (*As glycerin is not found in the pulp of a tooth or the protoplasmic contents of the tubules*, we fail to see anything conclusive in these experiments.) *The essayist did not succeed in diffusing chloride of zinc through the root of a tooth at all. . . . (So far no experiments have demonstrated the passage of a coagulating agent through the side of the root of a tooth without first depriving it of its coagulating property).*"

The writer of the paper alluded to in the foregoing paragraph never willingly notices adverse criticism, recognizing the fact that each individual has the right of private judgment, and further, he regards legitimate criticism as a valuable aid in the development of thought, even though it be, as it often is, unscientific.

The paragraph quoted under the pretence of being a digest, in fourteen lines, of a paper containing three months of careful work, is so thoroughly false that one is forced to the conclusion that it was either written by an ignoramus or one who purposely and maliciously gave a character to the paper it never possessed. As the new journal has never had the moral courage to give the name of its editors, it is impossible to understand the probable motive for the paragraph. The writer of it, if he had the mental capacity to know anything, should have understood the reason for the use of glycerin, as it is clearly explained in the text, and further, the tubes were not "*partially*" but thoroughly filled.

The statement that the writer of the original article was never able to "diffuse chloride of zinc through the root of a tooth" is *absolutely false* from beginning to end; and in proof of this we quote from the article: "In the specimens prepared for the microscope, *the evidence is positive to the trained eye that every tube is filled with coagulated organic matter, and this has been so frequently repeated, and with precisely the same results, that I have no hesitation in accepting it as a fact.*" It is true the writer failed to verify Dr. Kirk's experiment, but even this may not have been conclusive.

A journal that begins its career by making false statements can never hope to win the confidence of even its friends, much less the general profession. It has so far failed in making a respectable summary from the dental journals, and it is, therefore, not remarkable that some one on the editorial staff, in the February number, fails to understand the meaning of the word "digest." With this conclusion the reading members of the dental profession will fully agree.

